# Full-thickness macular hole formation in proliferative diabetic retinopathy

**DOI:** 10.1038/s41598-021-03239-2

**Published:** 2021-12-13

**Authors:** Mei-Chi Tsui, Yi-Ting Hsieh, Tso-Ting Lai, Chun-Ting Lai, Hsuan-Chieh Lin, Tzyy-Chang Ho, Chang-Hao Yang, Chung-May Yang, Lu-Chun Wang

**Affiliations:** 1 Department of Ophthalmology, National Taiwan University Hospital, Taipei, Taiwan; 2 Department of Ophthalmology, Eye Center, China Medical University Hospital, China Medical University, Taichung, Taiwan; 3 Department of Ophthalmology, National Taiwan University Hospital Hsinchu Branch, Hsinchu, Taiwan; 4 Department of Ophthalmology, College of Medicine, National Taiwan University, Taipei, Taiwan; 5 Department of Ophthalmology, National Taiwan University Hospital Yunlin Branch, No. 579, Sec. 2, Yunlin Rd., Douliou, Yunlin County Taiwan

**Keywords:** Eyelid diseases, Retinal diseases, Diabetes complications

## Abstract

Twenty-one consecutive patients (21 eyes) having proliferative diabetic retinopathy (PDR) and fibrovascular proliferation (FVP) with optical coherence tomography (OCT) available before and after full-thickness macular hole (FTMH) formation were retrospectively reviewed. Four types of FTMH formation pathways in PDR were identified and were quite different from those in idiopathic conditions. The activity, severity and locations of FVP varied in PDR eyes destined to develop FTMHs. Type 1 was characterized by epiretinal membrane (ERM) and/or vitreomacular traction (VMT) inducing foveoschisis, intraretinal cysts or foveal detachment, followed by formation of a FTMH or macular hole retinal detachment (MHRD). In type 2, ERM and/or FVP induced lamellar macular hole (LMH) with foveoschisis, followed by the formation of FTMH or MHRD. Type 3 was characterized by the initial tractional retinal detachment (TRD) with foveal cysts and/or foveoschisis and the subsequent formation of MHRD. Type 4 was characterized by TRD associated with foveal thinning, ensued by the formation of MHRD. The severity of FVP was grade 2 in 66.7% of eyes in both types 1 and 4, and grade 3 in 75% of eyes in type 3 while the severity of FVP was more evenly distributed in type 2.

## Introduction

A full-thickness macular hole (FTMH) most commonly appears in senile patients without apparent underlying diseases. It may also occur after blunt ocular trauma or in the condition of high myopia, rhegmatogenous retinal detachment (RD), or diabetic retinopathy^1–3^. The initial abnormalities in the formation of an idiopathic FTMH have been widely attributed to the persistent adherence of the cortical vitreous to the fovea with adjacent vitreoretinal separation. The resultant traction on the fovea may cause intraretinal cysts, focal outer retinal disruption or foveal detachment. Further traction may lead to dehiscence of the foveal tissue, resulting in FTMH formation^4–7^. Another mechanism involves an adherent posterior hyaloid itself, or the presence of epiretinal membrane (ERM) after vitreomacular separation; these tissues exert tangential traction on the fovea and generate a FTMH^8,9^. In eyes with proliferative diabetic retinopathy (PDR), unique pro-proliferative microenvironment exists and promotes ERM and fibrovascular proliferation (FVP) formation, causing complex traction on the macula^10–13^. Thus, the configuration of the macula and mechanisms of FTMH formation may be distinct from those in the idiopathic FTMHs. However, there is little research on mechanisms of FTMH formation in PDR. Our study group had briefly reported 4 cases of FTMH formation in PDR by observing optical coherence tomography (OCT) images^14^. In this study, we examine more cases and report the specific structural changes before and after FTMH formation by using color photos and OCT in patients with PDR. We attempt to elucidate the various pathways of FTMH formation in PDR.

## Materials and methods

Medical records were retrospectively reviewed on consecutive patients with known PDR who developed FTMHs at National Taiwan University Hospital during the period of November 2008 to March 2020. This study was approved by the Research Ethics Committee of National Taiwan University Hospital and was conducted in accordance with the Declaration of Helsinki. We identified 21 eyes of 21 patients having sequential OCT images before FTMH formation with a minimum follow-up duration of 4 months after the development of FTMH or after operation. All patients received serial OCT examinations and for each patient, the same OCT device was used if possible to avoid error induced by using different devices. RTVue XR or 100 (Optovue, Inc., Fremont, CA, USA) was conducted in 19 eyes (90.5%) and Cirrus HD-OCT (Carl Zeiss Meditec, Inc., Dublin, CA, USA) was used in 1 eye (4.8%). Only one patient who lost to follow-up before 2010 and another 3 patients in their earlier periods (before 2013) were performed with Stratus OCT (Carl Zeiss Meditec, Inc., Dublin, CA, USA). The OCT devices adopted standardized protocols and software for imaging and measuring to ensure repeated evaluation of the same point at different time frames. The horizontal and vertical scans of OCT were used for serial follow-up and comparison. Pre-FTMH, post-FTMH and post-operative ophthalmological examination results including best-corrected visual acuity (BCVA), intraocular pressure, lens status (phakic or pseudophakic), severity, activity (active or mainly fibrotic), extent, and locations of FVP, and the extent of RD were recorded. Relevant OCT parameters such as vitreomacular traction (VMT), the presence of ERM, foveoschisis, intraretinal cysts, foveal thinning, lamellar macular hole (LMH), foveal detachment, central macular thickness (CMT), macular hole (MH) edges (flat or elevated), and MH minimal and basal diameters were documented for analysis. The CMT was provided automatically by the OCT device whereas the MH minimal and basal diameters were manually measured. The OCT images were reviewed and analyzed independently by two of the authors (MCT and LCW). In case of doubt, the senior author (CMY) was consulted and a panel discussion was held to reach a consensus. In this study, ERM was defined as the presence of an irregular and hyperreflective layer over the internal limiting membrane (ILM). Foveal thinning was defined as the CMT (from ILM to above retinal pigment epithelium) measuring 200 μm or less. The diagnosis of LMH was made based on the criteria proposed by the International Vitreomacular Traction Study (IVTS) group^15^, which include an irregular foveal contour, a break in the inner retinal layer at fovea, separation between the inner and outer foveal retinal layers, and an absence of a full-thickness foveal defect with preservation of foveal photoreceptors. FTMH was defined as a full-thickness retinal defect in the macular area with or without surrounding subretinal fluid (SRF). A macular hole retinal detachment (MHRD) was defined as SRF below FTMH extending more than one-disc diameter. Edges of MH were defined as flat when MH edges were not elevated and contained minimal cystic spaces. MH minimal and basal diameters were measured on OCT scans. In cases with MHRD, only MH minimal diameters were measured. MH minimal diameter was defined as the shortest diameter of MH in an area excluding the operculum and could be in the outer or inner retina on OCT while basal diameter was defined as the length of the base of MH. FVP severity was classified into 4 grades based on the severity of vitreoretinal adhesion: multiple-point adhesions with or without 1-site plaque-like broad adhesion (Grade 1), broad adhesions in more than 1 but fewer than 3 sites, located posterior to the equator (Grade 2), broad adhesions in more than 3 sites, located posterior to the equator or extending beyond the equator within 1 quadrant (Grade 3), and broad adhesions extending for more than 1 quadrant anterior to the equator (Grade 4)^16^. Broad adhesion was defined as fibrovascular proliferative membrane having multiple point adhesions and occupying more than 2-disc areas^16^. The locations of FVP were divided into 5 categories^17^: (1) complete arcade type: FVP encompassed both upper and lower arcade vessels; (2) incomplete arcade type: FVP encompassed either upper or lower arcade vessels or their adjacent areas with or without disc involvement; (3) juxtapapillary type: FVP existed around the disc and nasal to the disc; (4) central type: FVP was seen mainly in the macular area; (5) widespread type: FVP involved upper and lower arcade vessels, macular area, and juxtapapillary area or extended beyond equator for more than 1 quadrant. The ‘macular area’ was defined as the area with a diameter of about 5.5 mm centered on the foveal depression.

### Surgical procedure

Surgeries were performed by using a standard three-port pars plana vitrectomy only when clinically indicated (when anatomical and functional improvements after operation were expected) and informed consents were obtained preoperatively. ERM and ILM in the macular areas were removed with or without an inverted ILM flap for premacular membrane which caused traction and structural changes. Visually significant cataracts were removed, and intraocular lenses were implanted when indicated. If necessary, supplementary panretinal photocoagulation (PRP) was done, and intravitreal injection of bevacizumab 1.25 mg was performed at the end of surgery.

### Statistical analysis

Means and standard deviations were calculated for quantitative variables. Frequencies and percentages were calculated for categorical variables. Mann–Whitney U test was used for comparing continuous variables between two groups, while Kruskal–Wallis analysis was used if there were more than two groups. For categorical data, either Fisher’s exact test or Chi square test was used. Wilcoxon signed-rank test was used for detecting differences between variables before and after FTMH formation. *P* < 0.05 was considered statistically significant. The statistical analysis of the data was done by the software Statistical Package for Social Sciences (SPSS) version 22.

## Results

### Demographics

Twenty-one eyes of 21 patients (6 male and 15 female) were analyzed in this study. Patient ages ranged from 29 to 69 years (mean: 51.1 ± 10.5 years). Two patients (9.5%) had type 1 diabetes mellitus (DM), and the other 19 patients (90.5%) had type 2 DM. The four most common comorbidities were hypertension, dyslipidemia, heart disease, and chronic kidney disease in 9 patients (9 eyes), 8 patients (8 eyes), 3 patients (3 eyes), and 3 patients (3 eyes), respectively. The average time duration from a prehole stage or the first clinic visit to FTMH detection was 19.1 ± 27.0 months (range: 0 to 100 months; median: 9 months). MHRD was found in 14 eyes (66.7%) whereas FTMH without RD was observed in 7 eyes (33.3%). The average MH minimal diameter of all eyes was 431 ± 206 μm. In eyes with MH and without RD, the average MH minimal diameter was 318 ± 122 μm and the average base diameter was 846 ± 449 μm. In eyes with MHRD, the average MH minimal diameter was 488 ± 219 μm. At the time of FTMH formation, 2 eyes (9.5%) had grade 4 FVP, 5 eyes (23.8%) had grade 3 FVP, 10 eyes (47.6%) had grade 2 FVP, and 4 eyes (19.0%) had grade 1 FVP, while 13 eyes (61.9%) had fibrotic FVP, and 8 eyes (38.1%) had active FVP. The locations of FVP were complete arcade type in 2 eyes (9.5%), incomplete arcade type in 13 eyes (61.9%), central type in 3 eyes (14.3%), and widespread type in 3 eyes (14.3%).

Before the formation of FTMHs, PRP was performed in 16 eyes (76.2%) and intravitreal injection of anti-vascular endothelial growth factor (anti-VEGF) was performed in 9 eyes (42.9%). One patient lost to follow-up after the diagnosis of FTMH was made. The average follow-up duration of the remaining 20 eyes was 44.8 ± 35.5 months (range: 4–117 months; median: 42 months). Spontaneous closure of FTMH was found in 3 of the 20 eyes (15.0%) with a duration of 2, 4 and 19 months, respectively. The other 17 eyes (85.0%) received vitrectomy for treatment of FTMH. Table 1 summarized the data for each eye in this study.

**Table 1:** Characteristics of each eye in this study.

Age	Gender	Laterality	SE^a^	Initial BCVA, logMAR (Snellen Equivalent)	Lens status	FVP severity	FVP locations	MH minimal diameter (μm)	MH basal diameter (μm)	MHRD	Type of MH formation	Late foveal contour	Final BCVA, logMAR (Snellen Equivalent)
55	F	R	2.25	1.301 (20/400)	Phakic	1	Central	456	1039	N	2	U	0.398 (20/50)
41	M	L	9.00	2 (20/2000)	Phakic	3	Widespread	239	N/A	Y	4	U	0.824 (20/133)
66	F	L	–	0.523 (20/67)	Pseudophakic	1	Central	259	594	N	2	U	0.824 (20/133)
63	F	R	3.00	1.9 (20/1589)	Phakic	4	Widespread	884	N/A	Y	2	W	2 (20/2000)
38	F	L	− 3.75	1.495 (20/625)	Phakic	2	Incomplete	640	N/A	Y	1	V	0.796 (20/125)
57	F	R	0.50	1.301 (20/400)	Phakic	2	Incomplete	632	N/A	Y	3	partially attached	2.4 (20/5024)
53	F	R	− 0.25	0.699 (20/100)	Phakic	2	Incomplete	213	213	N	1	U	0.523 (20/67)
39	F	R	− 1.00	2 (20/2000)	Phakic	2	Complete	710	N/A	Y	4		
54	M	L	2.00	1.301 (20/400)	Phakic	2	Incomplete	472	838	N	1	U	0.699 (20/100)
47	F	R	1.50	1.398 (20/500)	Phakic	3	Complete	610	N/A	Y	3	U	0.699 (20/100)
69	F	R	1.75	2.3 (20/3990)	Phakic	1	Central	136	N/A	Y	1	W	2.4 (20/5024)
53	F	L	− 0.25	1.398 (20/500)	Phakic	2	Incomplete	322	1778	Y	1	V	0.301 (20/40)
45	F	L	− 1.25	0.699 (20/100)	Phakic	2	Incomplete	173	482	N	2	U	0.398 (20/50)
59	M	R	0.75	1.699 (20/1000)	Phakic	2	Incomplete	164	N/A	Y	2	U	0.824(20/133)
29	F	R	− 2.00	0.824 (20/133)	Phakic	3	Incomplete	452	N/A	Y	3	V	0.201 (20/32)
43	F	L	− 1.00	1.301 (20/400)	Phakic	2	Incomplete	579	N/A	Y	4	W	0.398 (20/50)
40	M	L	− 1.50	0.699 (20/100)	Phakic	2	Incomplete	401	1296	N	2	U	2 (20/2000)
59	F	R	1.50	1.9 (20/1589)	Phakic	4	Widespread	598	N/A	Y	2	U	2 (20/2000)
59	F	L	2.25	1.9 (20/1589)	Pseudophakic	3	Incomplete	517	N/A	Y	3	W	2 (20/2000)
60	M	L	− 0.50	1 (20/200)	Phakic	1	Incomplete	252	1459	N	2	W	1 (20/200)
45	M	L	− 0.25	1.699 (20/1000)	Phakic	3	Incomplete	352	N/A	Y	1	W	2 (20/2000)

*BCVA* best-corrected visual acuity, *Complete* complete arcade type, *F* female, *FVP* fibrovascular proliferation, *Incomplete* incomplete arcade type, *M* male, *MH* macular hole, *MHRD* macular hole retinal detachment, *N* no (no MHRD), *N/A* not applicable, *SE* spherical equivalent, *Y* yes (MHRD).

^a^1/21 SE was not available.

### Structural changes of formation of FTMH

**Figure 1:**
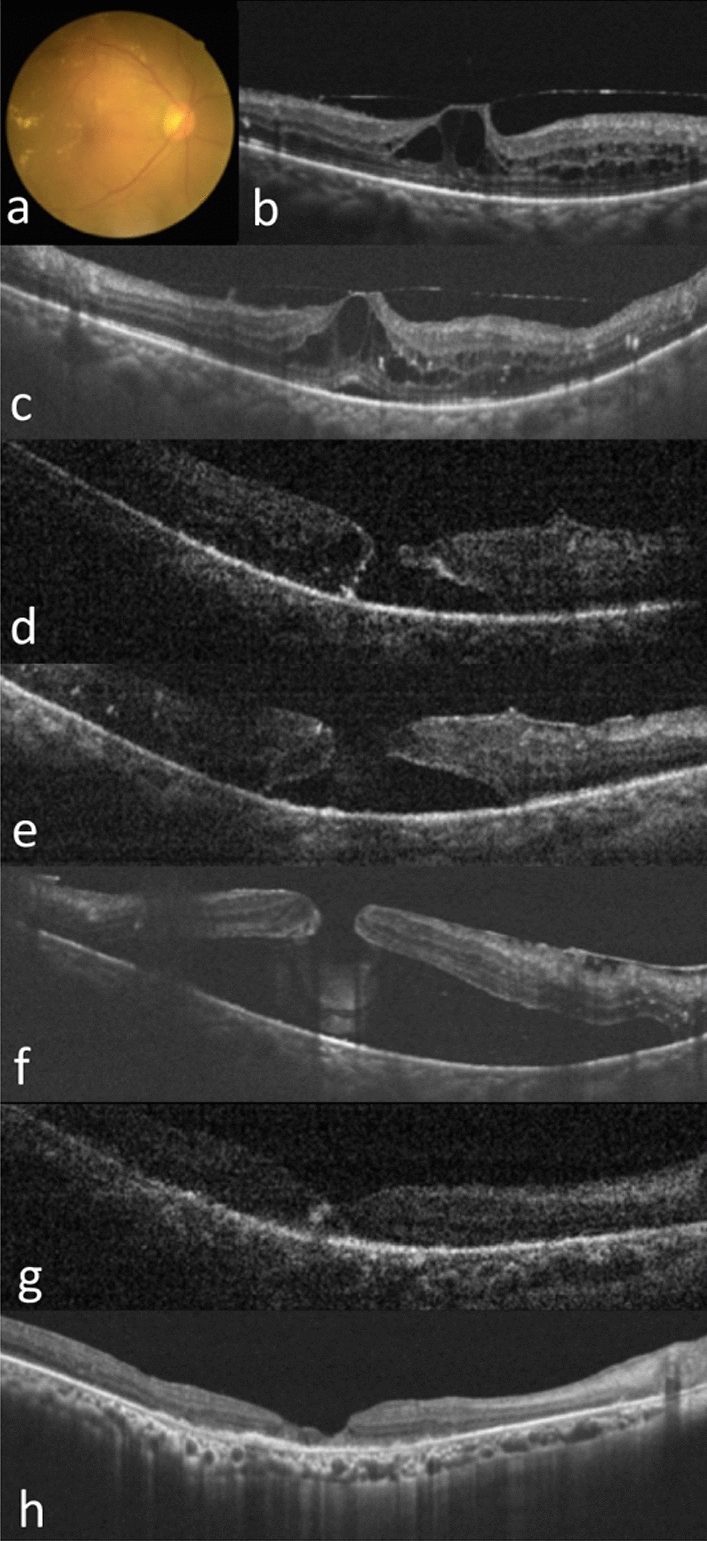
Type 1 pathway of full-thickness macular hole (FTMH) formation in the right eye of a 69-year-old woman with proliferative diabetic retinopathy (PDR). (**a**,**b**) Fundus photography and optical computed tomography (OCT) showed hard exudates temporal to the fovea and vitreomacular traction (VMT) with foveoschisis and foveal cysts. (**c**) 1 month later, foveal detachment with progression of foveoschisis and foveal cysts developed. (**d**) OCT showed a FTMH without the adhesion of posterior hyaloid 5 months later. (**e**) 1 month later, the FTMH and foveal detachment became larger, and epiretinal membrane (ERM) was visible. (**f**) Another 1 month later, macular hole retinal detachment (MHRD) was noted. (**g**) 2 weeks after surgery, U-shaped macular hole (MH) closure was noted. (**h**) 1.5 years later, foveal contour changed to W-shaped closure.

**Figure 2:**
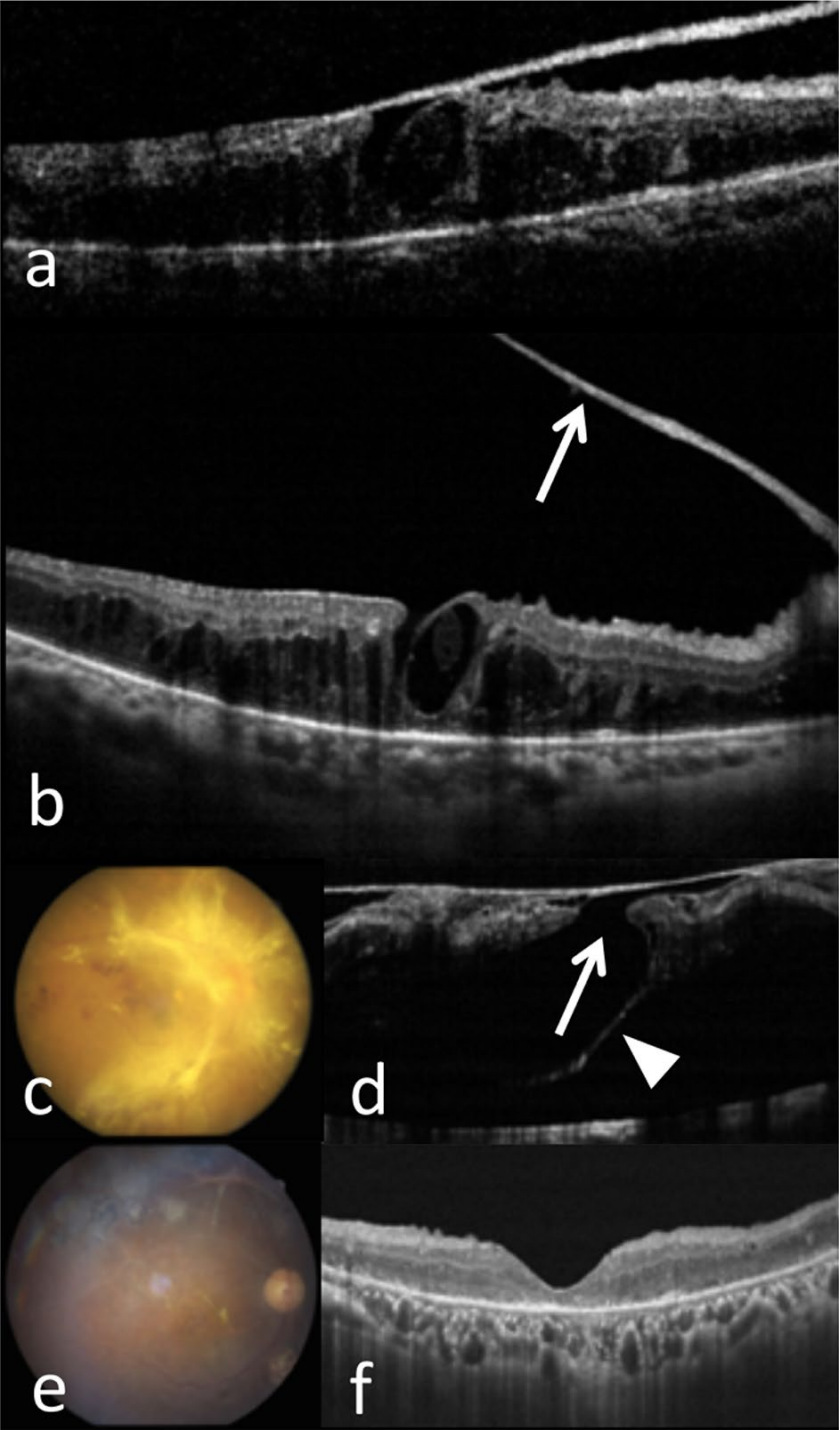
Type 2 pathway of FTMH formation in the right eye of a 59-year-old woman with PDR. (**a**) Lamellar macular hole (LMH) with foveoschisis induced by ERM and fibrovascular proliferation (FVP) was shown on OCT. (**b**) 1 year later, the ERM and FVP (arrow) separated from the fovea, but abnormal macular configuration persisted. (**c**,**d**) 1 month later, widespread type of FVP was noted on fundus photography, and a large FTMH (598 μm, arrow) developed. Subretinal fibrosis (arrowhead) was shown on OCT. (**e**,**f**) 1.5 years after surgery, the retina was attached on the fundus photography, and OCT showed U-shaped closure.

**Figure 3:**
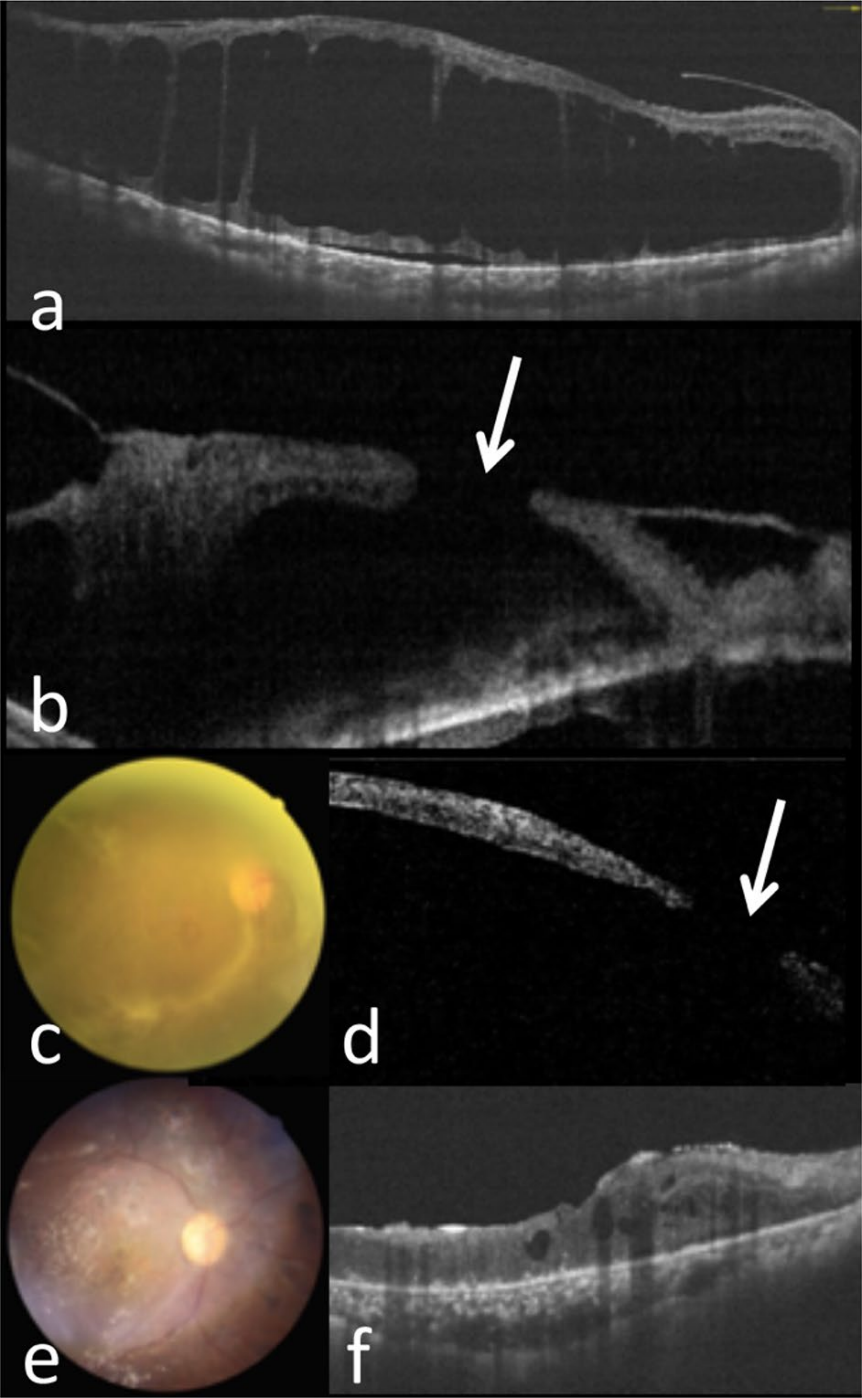
Type 2 pathway of FTMH formation in the right eye of a 63-year-old woman with PDR. (**a**) OCT showed tractional retinoschisis (TRS). (**b**) 1 year later, OCT showed TRS progression with LMH formation (arrow). (**c**,**d**) 1 month later, fundus photography revealed FVP mainly at inferior arcade and superotemporal macula; OCT showed MHRD (arrow) and flat hole edge without cystic changes. (**e**,**f**) 6 months after surgery, the retina was attached and the MH was closed; minor intraretinal cysts were noted.

Four different types of FTMH formation pathways in PDR could be identified. Type 1 was observed in 6 eyes (28.6%) and was characterized by ERM and/or VMT causing foveoschisis, intraretinal cysts or foveal detachment, followed by formation of a FTMH or MHRD (Fig. 1). Type 2 was found in 8 eyes (38.1%), and in this type, LMH with foveoschisis was the characteristic feature, which was induced by ERM (Fig. 2) or tractional retinoschisis (TRS) (Fig. 3) and it finally progressed into a FTMH or MHRD.

**Figure 4:**
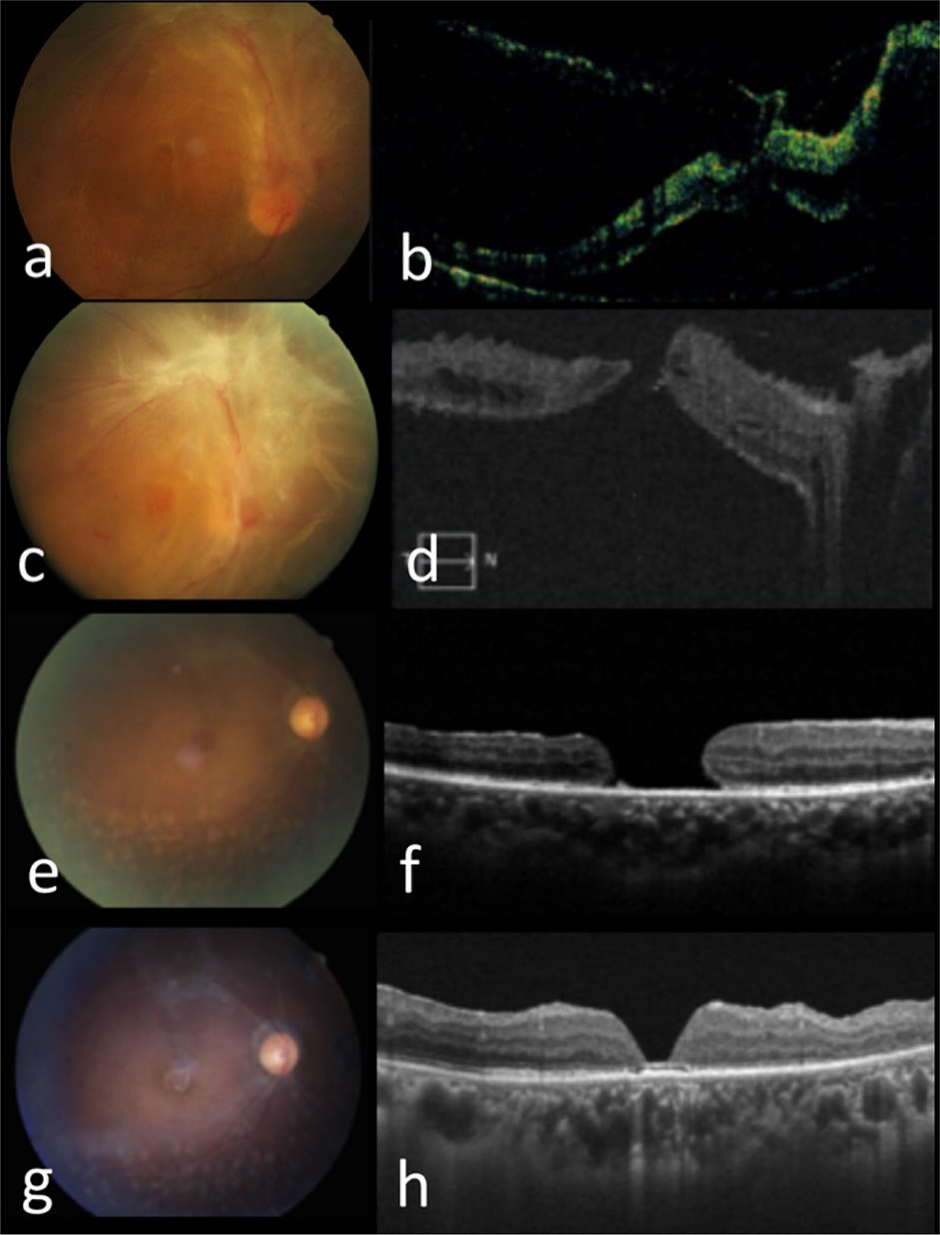
Type 3 pathway of FTMH formation in the right eye of a 29-year-old woman with PDR. (**a**) Fundus photography showed PDR with FVP located mainly on the upper retina (incomplete arcade type). (**b**) OCT showed TRD with foveoschisis. (**c**,**d**) 1 month later, FVP became more condensed on fundus photography, and MHRD (MH minimal diameter 452 μm) developed. (**e**,**f**) 4 years after operation, the retina was attached while a persistent MH enlarging gradually with time was revealed by OCT; vitrectomy with anterior lens capsule flap insertion was performed. (**g**,**h**) 4 months after surgery, the retina was attached and the FTMH was closed.

**Figure 5:**
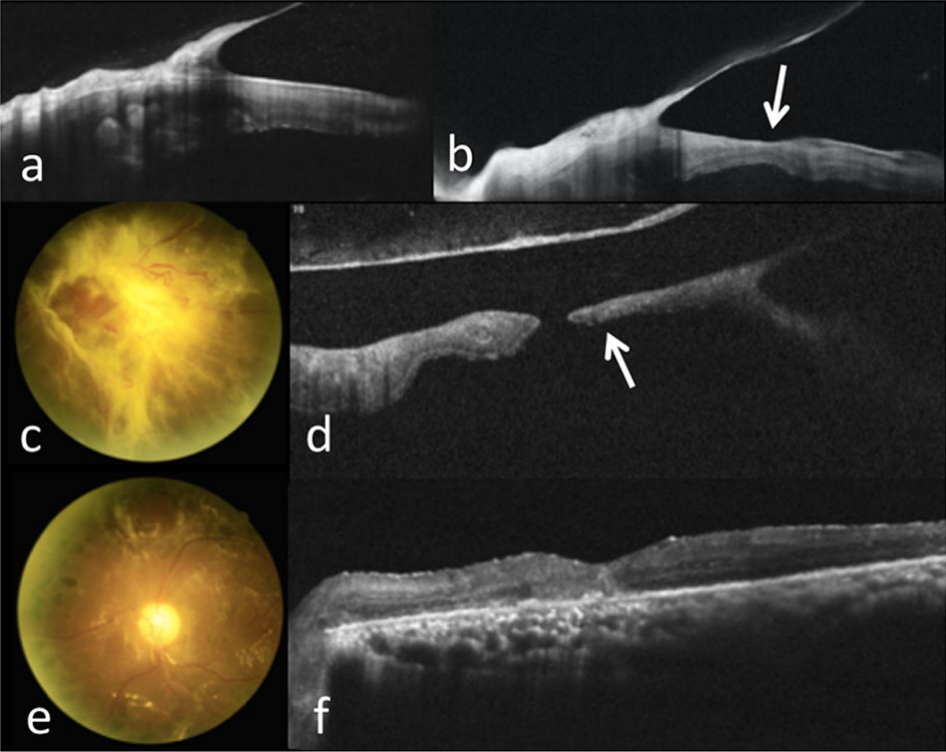
Type 4 pathway of FTMH formation in the left eye of a 41-year-old man with PDR. (**a**) TRD was noted on OCT. (**b**) 1 month later, TRD with mild foveal thinning (arrow) was detected on OCT. (**c**,**d**) 1 month later, fundus photography showed severe PDR with grade 4 FVP and MHRD (MH minimal diameter 239 μm) developed. Foveal thinning (arrow) was noted on OCT. (**e**,**f**) 1 year after surgery, fundus photography showed attached retina under silicone oil tamponade, and OCT revealed U-shaped MH closure.

Type 3 was detected in 4 eyes (19.0%), and was characterized by the initial tractional retinal detachment (TRD) with foveal cysts and/or foveoschisis and subsequent formation of MHRD (Fig. 4). Type 4 was noticed in 3 eyes (14.3%) and was characterized by TRD causing foveal thinning, ensued by the formation of MHRD because of persistent tractions (Fig. 5).

Different types of pathways were not statistically associated with age, gender, laterality, DM type, BCVA at the time of FTMH formation, lens status, MH size, MHRD, FVP severity, FVP activity, CMT, follow-up duration, final BCVA, early and final foveal contours; however, they were significantly associated with the presence of ERM/VMT (*p* = 0.001), TRD (*p* < 0.001), LMH (*p* < 0.001) and foveal thinning (*p* < 0.001). Predominantly more eyes in type 1 and type 2 groups had ERM/VMT than those in types 3 and 4. On the other hand, much more eyes in types 3 and 4 had TRD. Overall, the edges of FTMH were flat in 15 eyes (71.4%). Flat hole edges were observed in 5 eyes (83.3%), 5 eyes (62.5%), 2 eyes (50.0%), and 3 eyes (100.0%), in types 1, 2 (Fig. 3d), 3 and 4 (Fig. 5d), respectively. Locations, severity and activity of FVP varied in each case (Table 1). In type 1, the severities of FVP from grades 1 to 4 were 16.7%, 66.7%, 16.7% and 0% of eyes, respectively. In type 2, grades 1 to 4 were 37.5%, 37.5%, 25.0% and 0% of eyes, respectively. In type 3, 25.0% of eyes had grade 2 and 75.0% of eyes had grade 3 FVP. In type 4, 66.7% of eyes had grade 2 FVP and 33.3% of eyes had grade 3 FVP. The locations of FVP were incomplete arcade type (83.3%) and central type (16.7%) in type 1, incomplete arcade type (50.0%), followed by central type (25.0%) and widespread type (25.0%) in type 2, incomplete arcade type (75.0%) and complete arcade type (25.0%) in type 3, and complete arcade type (33.3%), incomplete arcade type (33.3%) and widespread type (33.3%) in type 4. Active FVP was found in 2 eyes (33.3%), 1 eye (12.5%), 2 eyes (50%), and 3 eyes (100.0%) in types 1, 2, 3 and 4, respectively. Two of the 3 eyes with spontaneous closure of FTMH belonged to type 2 FTMH formation pathway and FTMH closed spontaneously at 2 and 4 months, respectively. The other one presenting with MHRD belonged to type 1. In this case, the patient preferred not to have surgery, and during follow-up, the retina was reattached and spontaneous closure of FTMH was observed 19 months later. Although statistical investigation of the associated features was not possible because of the small number of cases, eyes with spontaneous closure of FTMH seemed to have better BCVA at the time of FTMH formation (logMAR 0.97 vs. logMAR 1.48) and smaller minimal MH diameter (261 μm vs. 460 μm) when compared to eyes without spontaneous closure of FTMH.

Eyes with MHRD were associated with poorer initial BCVA (LogMAR 1.67 vs. LogMAR 0.89, *p* < 0.001), shorter duration of FTMH formation (12.9 months vs. 31.7 months, *p* = 0.038), higher proportion of grade 3 and grade 4 FVP (50.0% vs. 0%, *p* = 0.030), higher proportion of active FVP (57.1% vs. 0%, *p* = 0.015), lower rate of ERM/VMT (42.9% vs. 100.0%, *p* = 0.015), and higher rate of TRD (50.0% vs. 0%, *p* = 0.030) when compared to those with FTMH and without RD. FVP severity was associated with its location (*p* < 0.001), and the presence of TRD (*p* = 0.047) as well as LMH (*p* = 0.043). Both of the 2 eyes with grade 4 FVP belonged to widespread type. All of the 3 eyes with central type FVP location had grade 1 FVP severity. Eyes with grade 3 FVP had predilection for TRD. Six of the 8 eyes (75.0%) with LMH had grade 1 or grade 2 FVP.

### Anatomical and functional outcomes

FTMHs were closed in 18 of the 20 eyes (90.0%) at three months after surgery or spontaneous closure, and in 20 eyes (100.0%) at the last follow-up. Retina was completely attached in 18 eyes (90.0%) and partially attached in 2 eyes (10.0%) at 3 months as well as at the last follow-up. Several foveal contours were noted at the last follow-up: U-, V-, W-shaped closure in 10 eyes (50.0%), 3 eyes (15.0%), and 6 eyes (30.0%), respectively, and partial retinal detachment in 1 eye (5.0%) (Table 1). BCVA improved from LogMAR 1.41 ± 0.53 to 1.15 ± 0.66 (*p* = 0.038) at 3 months postoperatively or 3 months after MH spontaneous closure and 1.13 ± 0.77 (*p* = 0.133) at the last follow-up. Thirteen eyes (65.0%) achieved a final visual acuity of 20/200 or better. BCVA improved or was stable in 17 eyes (85.0%) at the last follow-up.

## Discussion

In this study, we reported four different types of FTMH formation pathways in PDR. In type 1, the ERM or posterior hyaloid membrane causing vitreofoveal traction showed similar pattern to the prehole condition in idiopathic cases, except that the traction was contributed by multi-layered membranes. Therefore, unlike the idiopathic conditions in which traction is usually released after forming a gap in the inner cysts, traction persists even after separation of the posterior hyaloid from the fovea in PDR cases. The persistent traction exerted from FVP or ERM to the foveal area induced progressive foveoschisis and/or intraretinal cystic change, leading to FTMH or MHRD. One unique characteristic of the FTMH in PDR was that in 83.3% of cases the hole edge was flat, instead of being elevated with obvious intraretinal cysts. We speculated that the tangential traction, and not the anterior–posterior or oblique traction, played a more important role in the FTMH formation. In type 2, LMH with foveoschisis was the specific feature, which developed from tractional retinoschisis secondary to ERM and/or fibrovascular traction. This configuration was quite different from idiopathic LMH which was rarely associated with retinoschisis, and whose natural history was considered to be more stable^18^. Moreover, all eyes in this type had foveoschsis and/or intrareitnal cysts with tractional membrane, suggesting that the mechanism of LMH was a tractional rather than a degenerative process.

In type 3, TRD first developed by the severe oblique macular traction from the condensed hyaloid and FVP. However, instead of tension release after macular detachment, the nondetached vitreous hyaloid along with FVP tissue maintained a persistent tangential and anterior–posterior traction on the macula, causing disruption of the fovea and formation of MHRD. In our series, all eyes with this pattern developed MHRD instead of simple FTMH. The ultimate development of MHRD in this type emphasized the persistent and strong nature of the traction force in TRD. In type 4, there was TRD associated with foveal thinning before FTMH formation. One eye in this pattern presented with macula-on TRD initially and subsequent foveal thinning before FTMH formation. Foveal thinning may occur before or after TRD involving the macula. MHRD rather than the simple FTMH ensued in all cases in this type. We attributed the mechanism of foveal thinning mainly to the tractional force on the fovea, but ischemia and neurodegeneration might also play some roles. The inner retina was relatively hypoxic and more vulnerable to metabolic stress induced by diabetes, tissue degeneration or cell loss might lead to retinal thinning^19^. Apoptosis of neuroglial cells, induced by hyperglycemia and advanced glycation end products^20,21^ might also contribute to foveal thinning.

Locations, severity and activity of FVP varied in each case. In 66.7% of eyes, the FVP severities were either grade 1 or grade 2 at the time of FTMH formation. This observation indicated that FTMHs could develop even in eyes with mild FVP and mild PDR. The key factor was the traction force exerted on the fovea area. Further, FTMH formation might occur in either active (38.1%) or fibrotic (61.9%) stage of the disease. In the active stage, the traction force on the fovea might be getting stronger as the disease progressed, while in the fibrotic stage, the atrophic macula may contribute to the vulnerability of the structure against a milder traction. We observed at least 4 months after operation since our previous study showed most of the postoperative complications in PDR were managed within 4 months postoperatively^16^. The MH closure rate in this series was high compared with the previous studies^22^.

Improvements in surgical instruments and techniques may play important roles in increasing the MH closure rate, although case selection bias could not be ruled out. Compared to eyes with FTMH without RD, those with MHRD had poorer BCVA, shorter duration of FTMH formation, higher proportion of grade 3 and grade 4 FVP, higher rate of active FVP, lower rate of ERM/VMT, and higher rate of TRD, all these indicating that MHRD was more likely to occur in eyes with more severe PDR.

Our study is limited by the retrospective nature and the small number enrolled. However, in this series, we were able to identify four different types of FTMH formation pathways in PDR, which were quite different from those in idiopathic conditions. The foveal might be subject to multi-directional tractions by the ERM, posterior hyaloid, and/or fibrovascular membrane (types 1 and 2) or in the presence of TRD (types 2, 3 and 4), and these complex and strong tractions could induce retinal tears, LMH (type 2), and foveal thinning (type 4) either from the attached retina or after macular detachment. MHRD was more likely to occur in eyes with severe PDR. Spontaneous closure of FTMHs in PDR might be observed. The activity, severity and locations of FVP varied in PDR eyes destined to develop FTMHs. The understanding of the various configuration patterns of FTMH formation may help in guiding follow-up schedule, surgical planning and management. Whether our classification of MH formation pathways is adequate and thorough enough requires further study with a larger number of cases.
